# Correlation between mass and volume of collected blood with positivity of blood cultures

**DOI:** 10.1186/s13104-015-1365-8

**Published:** 2015-08-28

**Authors:** Lariessa Neves, Alexandre Rodrigues Marra, Thiago Zinsly Sampaio Camargo, Maura Cristina dos Santos, Flávia Zulin, Patrícia Candido da Silva, Natália Ariede de Moura, Elivane da Silva Victor, Jacyr Pasternak, Oscar Fernando Pavão dos Santos, Michael B. Edmond, Marines Dalla Valle Martino

**Affiliations:** Intensive Care Unit, Hospital Israelita Albert Einstein, São Paulo, Brazil; Instituto Israelita de Ensino e Pesquisa Albert Einstein, São Paulo, Brazil; Division of Medical Practice, Hospital Israelita Albert Einstein, Av. Albert Einstein, 627/701, 1st floor, Room 108, Bloco A1, Morumbi, São Paulo, 05651-901 Brazil; Department of Microbiology, Hospital Israelita Albert Einstein, São Paulo, Brazil; Department of Internal Medicine, Virginia Commonwealth University School of Medicine, Richmond, VA USA

**Keywords:** Blood culture, Bloodstream infection, Diagnosis, Automated methods, Infection, Quality indicator

## Abstract

**Background:**

The collection of blood cultures is an extremely important method in the management of patients with suspected infection. Microbiology laboratories should monitor blood culture collection.

**Methods:**

Over an 8-month period we developed a prospective, observational study in an adult Intensive Care Unit (ICU). We correlated the mass contained in the blood vials with blood culture positivity and we also verified the relationship between the mass of blood and blood volume collected for the diagnosis of bloodstream infection (BSI), as well as we explored factors predicting positive blood cultures.

**Results:**

We evaluated 345 patients with sepsis, severe sepsis or septic shock for whom blood culture bottles were collected for the diagnosis of BSI. Of the 55 patients with BSI, 40.0 % had peripheral blood culture collection only. BSIs were classified as nosocomial in 34.5 %. In the multivariate model, the blood culture mass (in grams) remained a significant predictor of positivity, with an odds ratio 1.01 (i.e., for each additional 1 mL of blood collected there was a 1 % increase in positivity; 95 % CI 1.01–1.02, p = 0.001; Nagelkerke R Square [R^2^] = 0.192). For blood volume collected, the adjusted odds ratio was estimated at 1.02 (95 % CI: 1.01–1.03, p < 0.001; R^2^ = 0.199). For each set of collected blood cultures beyond one set, the adjusted odds ratio was estimated to be 1.27 (95 % CI: 1.14–1.41, p < 0.001; R^2^ = 0.221).

**Conclusions:**

Our study was a quality improvement project that showed that microbiology laboratories can use the weight of blood culture bottles to determine if appropriate volume has been collected to improve the diagnosis of BSI.

## Background

The collection of blood cultures is crucial in the management of patients with suspected infection. It is the key piece of information in the etiologic diagnosis of septic shock, and for the choice of appropriate antimicrobial therapy [1, 2]. However, like any other laboratory test, there may be false positive or false negative results [1, 3, 4].

Some studies indicate the importance of the volume of blood collected in blood cultures, since the greater the collected volume of blood, the greater the rate of positivity, and thus the greater the detection rate of bloodstream infection [4–13]. The American Society for Microbiology (ASM) and the College of American Pathologists (CAP) recommends a collection volume of 30–40 mL for the diagnosis of bloodstream infection. This recommendation is based on observations made over 30 years ago, before the existence of automated blood culture systems [5, 6]. Thus, this study aims to correlate the mass contained in the blood vials with blood culture positivity and to verify the relationship between the mass of blood and blood volume collected for the diagnosis of bloodstream infection, as well as to explore factors predicting positive blood cultures.

## Methods

This study was a prospective, observational study conducted from December 2011 to July 2012 in the adult Intensive Care Unit of a tertiary hospital in the city of São Paulo, SP, Brazil.

Patients over 18 years old with sepsis, severe sepsis or septic shock were included in the study. This was a quality improvement study that was approved by the Institutional Review Board (IRB) of Hospital Israelita Albert Einstein. The requirement for informed consent was waived by our IRB in accordance of the Code of Federal Regulations and the Privacy Rule.

Sepsis was defined as infection plus two or more of the following SIRS criteria: T >38 or <36 °C; HR > 90/min; RR > 20 breaths/min (or PaCO_2_ < 32 mmHg); or WBC count >12,000 cells/μL or <4000 cells/μL (or >10 % band forms). Severe sepsis was defined as sepsis plus organ dysfunction, hypotension, or hypoperfusion abnormalities, including lactic acidosis, oliguria, or encephalopathy. Septic shock was defined as sepsis-induced hypotension (i.e., systolic BP <90 mmHg or a drop of >40 mmHg in the absence of other causes of hypotension) plus hypoperfusion abnormalities despite adequate fluid resuscitation [14]. Infections were classified as nosocomial if the patient was hospitalized more than 48 h when the culture was obtained [15].

If the bloodstream isolate was a potential skin contaminant (e.g., diphtheroids, *Propionibacterium* species, *Bacillus* species, coagulase-negative staphylococci, or micrococci), all of the following criteria were required for the diagnosis: the presence of an intravascular catheter, the initiation of targeted antimicrobial therapy, and at least one clinical finding (temperature >38.0 or <36 °C, chills, or systolic blood pressure of <90 mmHg [16, 17]. A blood culture contaminant was defined as a usual skin organism that was isolated from only one set of blood cultures in a patient with no evidence of an infection due to that organism [1, 18].

The results of blood cultures and clinical data, including age, gender, comorbidities, diagnosis, hospitalization, presence of bacteremia, and in-hospital mortality were collected. Any changes in antimicrobial therapy based on final results of blood cultures were also recorded.

The normal body temperature is about 37.0 °C (98.6 °F), but varies with the time of day and the measuring method used. The American College of Critical Care Medicine and the Infectious Diseases Society of America defines fever as an axillary body temperature above 38.3 °C (101 °F) [19]. Generally, in our hospital patients with an increase in body temperature to 37.8 °C have blood cultures collected per an automatic order in the patient chart. For many years, personnel have been trained to collect at least 10 mL for each blood culture bottle, to fill the bottle when possible, and to record the volume of blood obtained on the bottle.

Prior to use, blood culture bottles were weighed and then distributed for use in patients with suspected sepsis in the ICU, or in the emergency department for patients who were being admitted to the ICU. The mass in grams (g) of each vial was recorded on the bottle label. After blood collection was performed, the vials were sent to the microbiology laboratory, where they were weighed again and the weight recorded on the bottle label. The difference between the two measures corresponded to the blood mass collected.

Blood cultures were processed using BD BACTEC Plus Aerobic/F and Plus Anaerobic/F bottles and incubated in the BD BACTEC™ FX system for monitoring growth up to 5 days. Positive bottles were plated on CPS ID3 agar (bioMerieux), blood agar, and anaerinsol (Probac Brazil) for detection of aerobic and anaerobic bacteria. The identification of isolates was performed by manual and automated methods (XL Vitek, bioMerieux).

The microbiology laboratory has an alert system to notify physicians of patients with positive blood cultures and their gram stain results. Antimicrobial therapy was considered appropriate if the bacteria identified in the blood culture was susceptible to at least one of the antibiotics administered within 24 h after the collection of the culture. If the isolated microorganism was not susceptible by in vitro testing to the antibiotic used, the therapy was considered inadequate [20].

Finally, all the information was transcribed into a database, to correlate clinical data, blood mass, blood volume, positivity, and adequacy of antimicrobial therapy.

### Statistical analysis

The relationship between fever and blood culture positivity was assessed by Fisher’s exact test. Analysis of blood culture bottle factors and patient factors associated with positivity was performed by logistic regression models in simple and multiple approaches. The number of blood culture sets, total collected volume and total collected weight were not included simultaneously in the same model due to collinearity, so we adjusted three multiple models to evaluate their effects in the presence of confounders. Quality of adjustment was evaluated with Nagelkerke R Square. We used ROC curves to evaluate the predictive value of positive volume and weight. The area under the curve was estimated and accompanied by the 95 % confidence level. The analyses were performed with SPSS (SPSS Inc. SPSS Statistics 2008 for Windows, Version 17.0 Chicago: SPSS Inc.) and the level of statistical significance was set at 5 %.

## Results

We evaluated 345 patients with sepsis, severe sepsis or septic shock for whom blood culture bottles were collected for the diagnosis of bloodstream infection. Of these, 57 patients had blood cultures with growth of microorganisms, including 2 cases classified as contaminated and 55 cases of bloodstream infection. This resulted in a blood culture true positivity rate of 15.9 % (55/345).

Descriptive variables for the study population are shown in Table 1. The patients were predominantly male (62.6 %). The most common admission diagnoses were sepsis/septic shock/other shock states (45.2 %), followed by respiratory failure (20.9 %), neurologic disorders (15.1 %), and solid organ transplantation (6.4 %). A majority of the patients (53.9 %) had 3 or more comorbidities. The in-hospital mortality was 13.3 %.

**Table 1:** Characterisitics of the 345 septic patients with blood cultures obtained

	N	%
Male gender	216	62.6
Positive blood culture	55	15.9
Admission diagnosis		
Sepsis/septic shock/other shock state	156	45.2
Respiratory failure	72	20.9
Polytrauma	3	0.9
Neurologic disorders	52	15.1
Solid organ transplantation	22	6.4
Others	40	11.6
Number of comorbidities		
0	19	5.5
1	49	14.2
2	91	26.4
≥3	186	53.9
Number of collected blood culture sets		
1	80	23.2
2	92	26.7
3	78	22.6
4	33	9.6
≥5^a^	62	18.1
T ≥ 37.8 °C at time of collection	100	29.0
Source of the blood culture^b^		
Through a central venous catheter	12	3.5
Through a peripherically vein	185	54.6
Through a central venous catheter and peripherically vein	142	41.9
In-hospital mortality	46	13.3

^a^One case with 23 sets of blood culture

^b^There were 6 cases with no collection source identified

The number of blood cultures collected per patient ranged from 1 to 10, totaling 599 samples. Most patients had two blood culture sets (aerobic + anaerobic bottles) submitted (26.7 %). Twenty-three percent of patients (23.2 %) had one set, 22.6 % had 3 sets, 9.6 % had 4 sets, and 18.1 % had more than 5 sets (Table 1). Only 29.0 % had a temperature >37.8 °C at the time the blood cultures were collected. Blood culture bottles were more likely to have the initial weight recorded than the blood volume collected (91.6 % vs. 80.1 %, p < 0.001). Cultures were most commonly obtained via peripheral venipuncture (54.6 %); in 41.9 % cultures were obtained via peripheral venipuncture and through an existing central line, and in 3.5 % cultures were obtained only through an existing central line.

Table 2 shows that of the 55 patients with bloodstream infection, 40.0 % had peripheral blood culture collection only and 60.0 % had peripheral blood culture and central blood culture collection. There was a change in antibiotic therapy after the antibiogram became available in 12.7 %, and in 70.9 % of patients antimicrobial treatment was deemed appropriate. Bloodstream infections were classified as nosocomial in 34.5 %.

**Table 2:** Characteristics of the 55 patients with bloodstream infection

	N	%
Nosocomial infection	19	34.5
Change in treatment decision after gram stain reported	2	3.6
Change in treatment decision after antibiogram reported	7	12.7
Adequate antimicrobial treatment	39	70.9
Inadequate antimicrobial treatment	16	29.1
Blood culture collected via peripheral vein only	22	40.0
Blood culture collected via peripheral vein + central venous catheter	33	60.0
Monomicrobial infection	48	87.3
Polymicrobial infection	7	12.7

Among the patients studied, 87.3 % had monomicrobial infection and 12.7 % had polymicrobial infection (Table 3). Gram-positive bacteria were found in 43.6 % of patients, gram-negative bacteria in 36.4 %, and fungal infections in 7.3 %.

**Table 3:** Description of the microorganisms

	N = 55	%
Monomicrobial infection	48	87.3
Gram positive	24	50.0
*Staphylococcus epidermidis*	12	50.0
*Staphylococcus hominis*	4	16.7
*Staphylococcus haemolyticus*	2	8.3
*Staphylococcus aureus*	1	4.2
*Staphylococcus capitis*	1	4.2
*Staphylococcus sciuri*	1	4.2
*Streptococcus agalactiae*	1	4.2
*Clostridium difficile*	1	4.2
Identified as gram positive bacteria only	1	4.2
Gram negative	20	41.7
*Escherichia coli*	7	35.0
*Klebsiella pneumoniae*	4	20.0
*Pseudomonas aeruginosa*	4	20.0
*Neisseria meningitidis*	2	10.0
*Salmonella species*	2	10.0
*Serratia marcescens*	1	5.0
Fungi	4	8.3
*Candida albicans*	1	25.0
*Candida glabrata*	1	25.0
*Candida krusei*	1	25.0
*Cryptococcus neoformans*	1	25.0
Polymicrobial infection	7	12.7
*Klebsiella pneumoniae* + *Staphylococcus hominis*	1	14.3
*Candida albicans* + *Klebsiella pneumoniae*	1	14.3
*Candida albicans* + *Candida tropicalis*	1	14.3
*Enterococcus faecalis* + *Staphylococcus epidermidis*	1	14.3
*Klebsiella pneumoniae* + *Pseudomonas aeruginosa*	1	14.3
*Pseudomonas aeruginosa* + *Staphylococcus xylosus*	1	14.3
*Staphylococcus epidermidis* + *Escherichia coli*	1	14.3

Univariate analysis revealed that significant predictors of positive blood cultures were obtaining more than one set of blood cultures (OR: 1.28; CI 95 % 1.16–1.42; p < 0.001), body temperature (≥39.0 °C) (OR: 4.47; CI 95 % 1.16–17.21; p = 0.029), collected volume (OR: 1.02; CI 95 % 1.01–1.03; p < 0.001), and collected weight (OR: 1.02; CI 95 % 1.01–1.03; p < 0.001) (Table 4). In the multivariate model, which adjusted for age, gender, number of comorbidities, admission diagnosis and temperature >39 °C, the blood culture mass (in grams) remained a significant predictor of positivity, with an odds ratio 1.01 (i.e., for each additional 1 mL of blood collected there was a 1 % increase in positivity; 95 % CI 1.01–1.02, p = 0.001; Nagelkerke R Square [R^2^] = 0.192). For blood volume collected, the adjusted odds ratio was estimated at 1.02 (95 % CI: 1.01–1.03, p < 0.001; R^2^ = 0.199). For each set of collected blood cultures beyond one set, the adjusted odds ratio was estimated to be 1.27 (95 % CI: 1.14–1.41, p < 0.001; R^2^ = 0.221).

**Table 4:** Univariate analysis of factors predicting positive blood cultures

	Positivity, n (%)		OR	CI 95 %	P
	No	Yes			
Gender					
Female	118 (91.5)	11 (8.5)			
Male	172 (79.6)	44 (20.4)	2.74	1.36–5.53	*0.005*
Diagnosis on admission					
Sepsis/septic shock/other shock states	123 (78.8)	33 (21.2)			0.063
Respiratory failure	68 (94.4)	4 (5.6)	0.22	0.08–0.65	0.006
Neurologic disorders	46 (88.5)	6 (11.5)	0.49	0.19–1.24	0.130
Solid organ transplantation	18 (81.8)	4 (18.2)	0.83	0.26–2.62	0.748
Others	35 (81.8)	8 (18.6)	0.85	0.36–2.01	0.715
Median age (in years)^a^	68 (52–82)	68 (56–78)	1.00	0.98–1.01	0.884
Number of comorbidities^a^	3 (2–4)	3 (2–4)	1.00	0.82–1.22	0.972
Number of blood cultures pairs^a^	2 (1–3)	3 (2–7)	1.28	1.16–1.42	*<0.001*
Mean body temperature (°C) at time of collection^a^	36.7 (36.1–37.3)	37.0 (36.0–37.6)	1.20	0.90–1.62	0.220
Body temperature ≥37.8 °C at time of collection					
No	212 (86.5)	33 (13.5)			
Yes	78 (78.0)	22 (22.0)	1.81	1.00–3.30	0.052
Body temperature ≥39.0 °C at time of collection					
No	285 (84.8)	51 (15.2)			
Yes	5 (55.6)	4 (44.4)	4.47	1.16–17.21	*0.029*
Collected median volume (mL)^a^	35 (20–52)	56 (34–95)	1.02	1.01–1.03	*<0.001*
Collected median weight (g)^a^	35 (21–53)	54 (32–82)	1.02	1.01–1.02	*<0.001*

Italic values indicate statistically significant associations

^a^Data described by median and interquartile range (first quartile–third quartile)

**Fig. 1:**
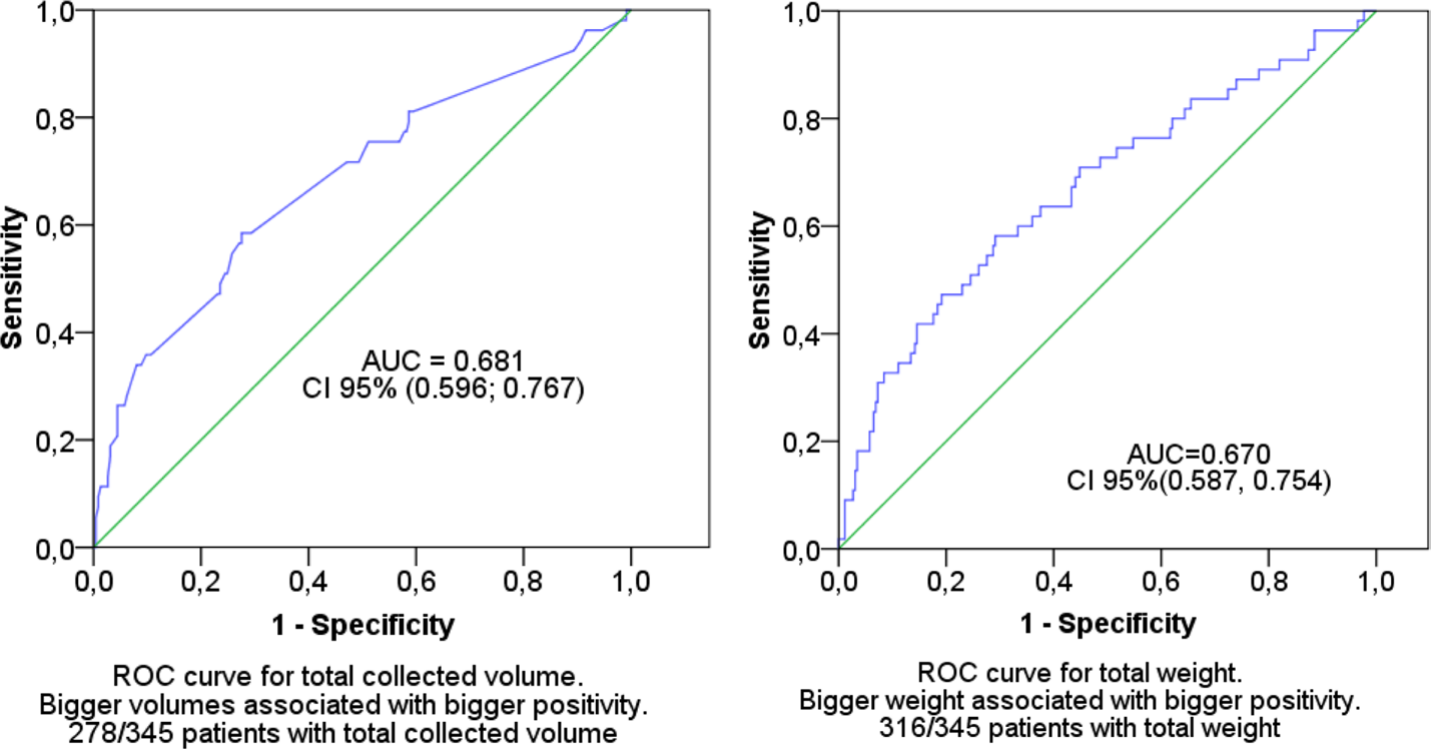
Relationship between total collected blood volume or total weight and positivity of blood cultures. On the *left* ROC curve for total collected volume; on the *right* ROC curve for total weight. There is evidence between the total collected volume or total weight and positivity of blood cultures when analysed per patient

There is strong evidence of a relationship between the total collected volume or total weight and positivity of blood cultures when analyzed per patient as seen in Fig. 1.

## Discussion

Blood cultures remain an important laboratory test in patients with systemic inflammatory response syndrome. The number of blood cultures collected in this study ranged from 1 pair to 23 sets, always including an aerobic and an anaerobic bottle. However, more recent studies recommend collecting at least 2 and at most 4 sets of blood culture per infectious episode. Collection of more than three samples may delay initiation of empiric antimicrobial therapy [5, 6].

Collection of blood cultures from peripheral veins is preferred. Of the 55 patients with positive blood cultures, 60 % had blood cultures obtained via peripheral vein and from a central venous catheter at same time. The rate of blood culture contamination is approximately 10 % higher when blood is collected from an indwelling catheter [1, 5, 6].

Contamination of blood cultures is a relatively common occurrence in clinical practice. Of the 57 patients with positive blood cultures, 2 were determined to have contaminated cultures. Published criteria can help to identify contaminated cultures, such as the number collected and number positive, the identity of the microorganism, the time to positivity, the evolution of clinical signs and laboratory data, as well as information from automated methods [1, 5, 6].

The volume of blood is an important variable since the larger the volume of blood obtained, the greater the positivity rate. The appropriate volume of blood depends on the recommendation of the manufacturer and the system used by each health institution, however the recommendation of recent studies and surveys is 10 mL per bottle [5, 6, 13].

Although the volume of collected blood is the most important factor for the positivity of blood cultures [5, 6] and appropriate collection volumes can improve the diagnosis of bloodstream infection, it is difficult to ascertain that an adequate volume is collected in each bottle. Therefore, we decided to determine the weight of the collected blood for the diagnosis of bloodstream infection. To weigh pre- and post-blood culture is a somewhat laborious procedure, but it was considered more reliable. It was necessary to weigh prior to blood culture collection because we observed that the blood culture bottles had different weights. We proved that this variable can be used as a quality indicator for the microbiology laboratory because it is easier to measure than the exact volume collected in each bottle. Ultimately, improving the diagnosis of bloodstream infection contributes to appropriate antimicrobial therapy [21].

Currently, the indiscriminate use of antibiotics is causing microorganisms to become increasingly resistant to many agents, creating a serious worldwide problem [22, 23]. Patients who receive initial inappropriate antibiotics but then are adjusted when susceptibility testing becomes available also have improved outcomes. Adjusting therapy can decrease the antimicrobial spectrum and reduce the appearance of resistant microorganisms [23]. A high proportion of the patients in our study received appropriate antimicrobial therapy (more than 70 %). One possible explanation is that the microbiology laboratory notifies the physicians of patients with positive blood cultures and their gram stain results 24 h per day. Except for temperatures >37.8 °C, the elevated body temperatures were not associated with blood culture positivity, but for temperatures higher than 39 °C there was a statistically significant difference in blood culture positivity; however, the small number of cases do not permit of us to make any other conclusions.

Our study has some limitations. First, this study was performed at a single medical center so it may not be generalizable to all hospitals. Second, we limited our analysis of adequate antimicrobial therapy only to patients with confirmed bloodstream infections (positive blood cultures). We did not analyze adequate antimicrobial therapy for the patients with suspected infection and negative blood cultures.

## Conclusions

In conclusion, our study was a quality improvement project that showed that microbiology laboratories can use the weight of blood culture bottles to determine if appropriate volume has been collected to improve the diagnosis of bloodstream infection. Since volume and weight are correlated, measuring weight is a way to follow the recommendations of the College of American Pathologists.

## Abbreviations

ICU: Intensive Care Unit
BSI: bloodstream infection
ASM: American Society for Microbiology
CAP: College of American Pathologists
IRB: Institutional Review Board
